# The Prognostic Significance of Tumor Deposit Count for Colorectal Cancer Patients after Radical Surgery

**DOI:** 10.1155/2020/2052561

**Published:** 2020-03-17

**Authors:** Kuo Zheng, Nanxin Zheng, Cheng Xin, Leqi Zhou, Ge Sun, Rongbo Wen, Hang Zhang, Guanyu Yu, Chenguang Bai, Wei Zhang

**Affiliations:** ^1^Department of Colorectal Surgery, Changhai Hospital, Shanghai, China; ^2^Department of Pathology, Changhai Hospital, Shanghai, China

## Abstract

**Background:**

The prognostic value of tumor deposit (TD) count in colorectal cancer (CRC) patients has been rarely evaluated. This study is aimed at exploring the prognostic value of TD count and finding out the optimal cutoff point of TD count to differentiate the prognoses of TD-positive CRC patients.

**Method:**

Patients diagnosed with CRC from Surveillance, Epidemiology, and End Results (SEER) database from January 1, 2010, to December 31, 2012, were analyzed. X-tile program was used to identify the optimal cutoff point of TD count in training cohort, and a validation cohort was used to test this cutoff point after propensity score matching (PSM). Univariate and multivariate Cox proportional hazard models were used to assess the risk factors of survival.

**Results:**

X-tile plots identified 3 (*P* < 0.001) as the optimal cutoff point of TD count to divide the patients of training cohort into high and low risk subsets in terms of disease-specific survival (DSS). This cutoff point was validated in validation cohort before and after PSM (*P* < 0.001, *P* = 0.002). More TD count, which was defined as more than 3, was validated as an independent risk prognostic factor in univariate and multivariate analysis (*P* < 0.001).

**Conclusion:**

More TD count (TD count ≥ 4) was significantly associated with poor disease-specific survival in CRC patients.

## 1. Introduction

Colorectal cancer (CRC) is one of the most common malignancies worldwide. The number of CRC cases ranks third in malignancies worldwide, while the number of deaths caused by CRC ranks second [1, 2]. CRC not only is a leading cause of cancer-related death in developed countries but also gradually brings a huge health burden to developing countries [3]. Radical surgery is the only curative treatment for CRC patients. Staging of CRC is one of the cornerstones to determine postoperative prognosis and criteria for postoperative therapy. The American Joint Committee on Cancer (AJCC)/TNM staging system, which classifies the extent of cancer based on anatomic information, is the most commonly used staging scheme in CRC [4]. Site-specific factors, such as tumor deposit (TD), perineural invasion (PNI), and surgical resection margin, are also predictive factors of the outcomes of CRC patients [4]. TD, as one of the important site-specific factors, was first introduced in the 5th edition of AJCC/TNM staging system in 1997 [5]. Thereafter, several editions of AJCC/TNM staging manual have defined TD, which has allowed for more accurate prognostication and for refining therapy for CRC patients [6]. Since 7th AJCC/TNM staging system, TDs was introduced into N1c category in CRC patients without metastatic lymph nodes, and positive TD status has been shown to be associated with poor outcomes [7, 8]. However, unlike metastatic lymph nodes, only TD status, instead of TD count, was introduced into the staging system, which may lose useful prognostic information. On the other hand, the impact of TD count on outcomes of CRC patients has been rarely evaluated to date. In this study, we used the Surveillance, Epidemiology, and End Results (SEER) Program database to evaluate the prognostic value of TD count for TD-positive CRC patients after radical surgery and find optimal cutoff point of TD count to better distinguish the patients with worse outcomes from those with favorable outcomes.

## 2. Materials and Methods

### 2.1. Data Source

The dataset in this study was obtained from SEER database and selected by SEER-stat software (SEER^∗^Stat 8.3.5). The number of our permission to access the database was 15688-Nov2018. This study was approved by the Institutional Review Board of Changhai Hospital, Secondary Military Medical University, Shanghai, China.

### 2.2. Patient Selection

From the total cases of CRC diagnosed from 2010 to 2012, we included cases with the following characteristics: (1) patients were between 18 and 75 years old; (2) patients were TD-positive; (3) the TD count was clear; (4) tumor was located at the rectum, left colon, and right colon; (5) radical operation was performed; (6) CRC was the first and only malignant tumor; (7) neoadjuvant therapy was not taken; (8) pathological result was adenocarcinoma, mucinous adenocarcinoma, or signet-ring cell carcinoma; (9) survival was between 1 month and 30 years after surgery; and (10) clinicopathologic data were sufficient. After considering the above criteria, a total of 1252 patients were finally included in our study.

### 2.3. Data Collection

Multiple clinicopathologic characteristics were collected, including TD count, age, gender, race, tumor location, differentiation degree, pathological type, tumor size, T stage, the number of metastatic lymph nodes, M stage, carcinoembryonic antigen (CEA) level before surgery, and perineuronal invasion (PNI) status. Tumor deposits (TD) of colon cancer and rectum cancer are defined as one or more satellite peritumoral nodules in the pericolorectal adipose tissue of a primary carcinoma without histologic evidence of residual lymph node in the nodule, which conforms to the description in the 8th AJCC/TNM staging system [9] and the college of American Pathologist cancer protocol (v4.0.01.0) [10]. The TNM classification was evaluated according to the 8th AJCC/TNM staging system. The end point of this study was disease-specific survival (DSS) according to specific codes provided by SEER.

### 2.4. Statistical Analysis

Patients were divided randomly into two cohorts, the training cohort and the validation cohort, using computer-generated random numbers. The cutoff points of TD count were determined using the X-tile program in the training cohort, which identified the optimal cutoff value according to the minimum *P* values from log-rank chi-square statistics for the categorical TD count in terms of DSS, and the cutoff point was validated in the validation cohort. Survival curves were generated using Kaplan-Meier analysis, and the differences were analyzed by log-rank test. Clinicopathologic characteristics were compared using chi-square tests or Fisher's exact test for categorical variables and nonparametric test for hierarchical variables. A propensity score matching (PSM) was performed to reduce the possibility of selection bias by using a logistic regression model. All the clinicopathologic variables were used in matching. Patients were matched in a ratio of 1 : 2, and the patients could be repeatably matched. The caliper used for matching was set at 0.05.

All statistical analyses were performed using SPSS version 22.0 (IBM, New York, NY) and R statistical software (R, version 3.5.0; R Project). The difference was considered statistically significant for both sides (*P* value < 0.05).

## 3. Results

### 3.1. The Characteristics of Patients from SEER Database

A total of 1252 patients were included in this study, including 685 (54.6%) males and 567 (45.4%) females. The median age was 59 years old (range, 18-75 years). The median TD count was 2, with a range of 1 to 38. The median follow-up of all the patients was 46 months, with a range of 1 month to 71 months.

### 3.2. The Optimal Cutoff Value for TD Count in TD-Positive CRC Patients

To identify and validate a cutoff for the TD count, the 1252 patients were divided randomly into two cohorts, the training cohort and the validation cohort, using computer-generated random numbers. As shown in Table 1, no significant difference was shown in age, gender, race, tumor location, differentiation degree, pathological type, tumor size, T stage, the number of metastatic lymph nodes, M stage, CEA level, PNI status, and TD count between the training and validation cohorts (all *P* > 0.05).

**Figure 1:**
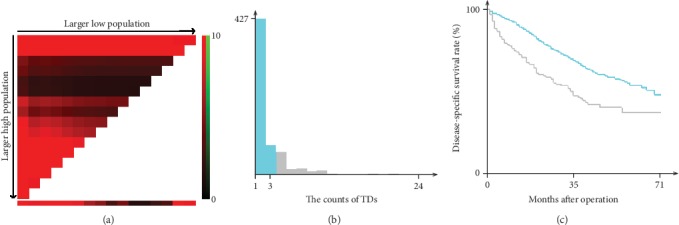
Division of patients in training cohort by the optimal cutoff point for the TD counts produced by X-tile plot. The optimal cutoff point highlighted by the black circle (a) is shown on a histogram of the training cohort (b) and a Kaplan-Meier plot (c). *P* values were determined using the cutoff point defined in the training cohort and applying it to the validation set. (The optimal cutoff point for TD count is 3, *χ*^2^ = 21.756, *P* < 0.001.).

Using the X-tile software, a cutoff point of TD count of 3 was yielded to distinguish the patients with poor outcomes and favorable outcomes in training cohort (*P* < 0.001, *χ*^2^ = 21.756, Figure 1). Using the same cutoff of TD count, 152 (24.6%) patients had more than 3 TDs, and 456 (75.4%) had 1-3 TDs. As showed in Table 2, TD count was significantly associated with differentiation degree (*P* < 0.05), the number of metastatic lymph nodes (*P* < 0.05), M stage (*P* < 0.05), and PNI status (*P* < 0.05) in both training cohort and validation cohort. Specifically, patients were more likely to be with higher differentiation degree, more metastatic lymph nodes, higher M stage, and more PNI in patients with more than 3 TDs.

### 3.3. Prognostic Value of TD Count in TD-Positive CRC Patients

**Figure 2:**
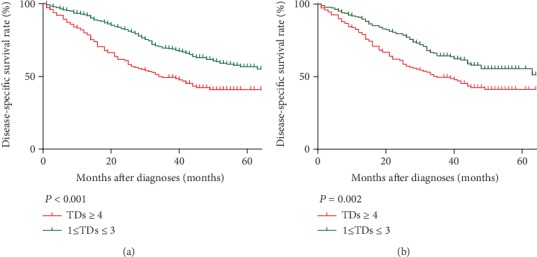
Log-rank tests of DSS comparing patients with TDs (1-3 vs. ≥4) before (a) and after (b) PSM.

In order to validate the cutoff point got from training cohort, we further compared the difference of DSS between patients with 1-3 TDs and patients with more than 3 TDs. As showed in Figure 2(a), patients with more than 3 TDs had significantly worse prognoses than patients with 1-3 TDs in the validation cohort (*P* < 0.001). The median follow-up time of this cohort was 38.5 months (range: 1-71 months). The 5 years of DSS rate was 41.0% (32.6%-49.4%) in patients with more than 3 TDs and 55.1% (47.5%-62.7%) in patients with 1-3 TDs. The median DSS time of patients with more than 3 TDs was 34 months while median DSS time of patients with 1-3 TDs was more than 71 months in validation cohort.

To reduce the possibility of selection bias, PSM was implemented in validation cohort. As shown in Table 2, PSM produced 188 patients with 1-3 TDs and 151 patients with more than 3 TDs. There was no significance in any variable between two groups. Kaplan-Meier DSS curves of two groups after PSM are shown in Figure 2(a). There was a statistically significant difference between DSS of the two groups (*P* = 0.002).

A Cox regression model is displayed in Table 3 to analyze the prognostic value of TD count in the PSM cohort. Univariate analysis showed that some clinicopathologic features, including tumor location (left colon vs. rectum, HR = 1.302, *P* = 0.236; right colon vs. rectum, HR = 2.378, *P* < 0.001), differentiation degree (HR = 1.640, *P* = 0.004), tumor size (HR = 1.444, *P* = 0.022), T stage (HR = 4.296, *P* = 0.013), the number of metastatic lymph nodes (1-3 vs. 0, HR = 1.114, *P* = 0.641; ≥4 vs. 0, HR = 2.041, *P* = 0.001), M stage (HR = 4.677, *P* < 0.001), CEA level (HR = 3.787, *P* < 0.001), PNI status (HR = 1.692, *P* = 0.001), and TD count (HR = 1.641, *P* = 0.002), were significant prognostic factors for DSS. However, multivariate Cox regression analysis indicated that only differentiation degree (HR = 1.640, *P* = 0.004), M stage (HR = 3.343, *P* < 0.001), CEA level (HR = 2.277, *P* < 0.001), and TD count (HR = 1.820, *P* < 0.001), were significantly associated with patients' survival prognosis, revealing that they were independent prognostic factors (all *P* < 0.05).

## 4. Discussion

After being defined by several editions of AJCC staging manual, TD was newly defined as isolated tumor foci in the pericolorectal fat or adjacent mesocolic fat away from the leading edge of the tumor without histological evidence of residual lymph node or identifiable vascular or neural structures. Shen et al. [11] reported that the cause-specific survival rate of TD-positive CRC patients was significantly worse than those of patients without TDs in the absence of metastatic lymph nodes. Bouquot et al. [12] reported that the disease-free survival rate was significantly worse for TD-positive patients compared to those without TDs. It was clear that positive TD status was an independent risk factor of poor prognosis of CRC without metastatic lymph nodes [6], and the classification of N1c has been introduced into AJCC TNM stage system [13]. In the CRC patients with metastatic lymph nodes, the prognostic value of TD status was neglected in AJCC staging system, which aroused worldwide discussion [7, 14, 15]. Mayo et al. [16] showed that TDs were associated with worse 3-year OS overall survival in patients of any known and unknown N categories, which suggested that TDs might be associated with a risk of all-cause death or cancer-specific death at least similar to a positive lymph node in all N categories. Basnet et al. [17] signified that TD was an independent prognostic factor associated with metastatic diseases along with vascular invasion and the number of metastatic lymph nodes among CRC patients. All these studies indicated that it might be more reasonable to differentiate prognostic significance of TD status from that of metastatic lymph nodes [18], and more details of TDs should be explored in CRC patients.

In this study, we evaluated the predictive role of TD count in TD-positive CRC patients after radical surgery. Using the X-tile software, we found that 3 is the optimal cutoff point of TD count to distinguish the patients with poor outcomes and favorable outcomes in training cohort. We further validated this cutoff point in a validated cohort. Considering these correlations might conceal the independent prognostic value of more TDs (TD count ≥ 4); we conducted PSM to get a PSM cohort from validation cohort with balanced baseline between more TDs (TD count ≥ 4) and less TDs (1 ≤ TD count ≤ 3). In PSM cohort, DSS was significantly worse for patients with more TDs (TD count ≥ 4) compared to those with less TDs (1 ≤ TD count ≤ 3). In multivariate analysis, we found that more TD count (TD count ≥ 4) was an independent prognosis for TD-positive CRC patients after radical surgery.

Several studies found that TDs occurred more frequently in cases with PNI [19, 20] and lymphatic invasion [6, 21]. The presence of TDs was also associated with higher T and M stages [6, 14, 22, 23]. Similar to those former studies, we demonstrated that more TD count (TD count ≥ 4) was associated with higher differentiation degree, more metastatic lymph nodes, higher M stage, and positive PNI, which implied that more TD count was an indicator of aggressive tumor biology and advanced stage.

This study has several noteworthy limitations. First, even though there was a clear definition of tumor deposit in the SEER database, however, the lack of definition of tumor deposit count might result in the heterogeneity in the practice of pathologist. Neither the 8th AJCC/TNM Manual nor the college of American Pathologist cancer protocol (v4.0.1.0) contains the accurate description of the tumor deposit count. Thus, we called on setting uniform standards in the evaluation of tumor deposit count. In our center, size and shape of the tumor focus were not regarded as the factors affecting the identification of a tumor deposit, which conforms to the College of American Pathologist Cancer Protocol (v4.0.1.0). To keep the evaluation of tumor deposit count reliable, one sample was usually assessed by two pathologists, respectively. The senior pathologist would settle the disputes if they occurred. Second, incomplete demographic and clinical information, especially TD count, in SEER database limited the inclusion of a larger number of patients. Third, the cutoff point of tumor deposit count got from this study needs to be further validated by the data from other center.

In conclusion, our SEER-based population study confirmed that TD count was an independent prognostic factor for CRC patients with positive TD status. Our data suggested TD count should be recorded in the pathology report routinely, and patients with more TD count (TD count ≥ 4) were recommended to received aggressive treatment after radical surgery.

## Figures and Tables

**Table 1:** Comparison of baseline characteristics of colorectal cancer between training cohort and validation cohort.

Characteristic	Training cohort (*n* = 635)	Validation cohort (*n* = 617)	*P* value
Age at diagnosis^a^ (years)	59 (18-75)	59 (26-75)	0.492
Gender			0.962
Male	347 (54.6)	338 (54.8)	
Female	288 (45.4)	279 (45.2)	
Race			0.356
White	502 (79.1)	467 (75.7)	
Black	69 (10.9)	76 (12.3)	
Other	64 (10.1)	74 (12.0)	
Tumor location			0.184
Rectum	122 (19.2)	120 (19.4)	
Left colon	273 (43.0)	293 (47.5)	
Right colon	240 (37.8)	204 (33.1)	
Differentiation degree			0.170
Well/moderately differentiated	473 (74.5)	480 (77.8)	
Poorly differentiated/undifferentiated	162 (25.5)	137 (22.2)	
Pathological types			0.799
Adenocarcinoma	585 (92.1)	566 (91.7)	
Mucinous adenocarcinoma/signet-ring cell carcinoma	50 (7.9)	51 (8.3)	
Tumor size (cm)			0.355
<5	350 (55.1)	324 (52.5)	
≥5	285 (44.9)	293 (47.5)	
T stage			0.193
T1/T2	34 (5.4)	44 (7.1)	
T3/T4	601 (94.6)	573 (92.9)	
The number of LNMs			0.146
0	166 (26.1)	191 (31.0)	
1-3	236 (37.2)	222 (36.0)	
≥4	233 (36.7)	204 (33.1)	
M stage			0.161
M0	400 (63.0)	412 (66.8)	
M1	235 (37.0)	205 (33.2)	
CEA level (U/ml)			0.510
<5	258 (40.6)	262 (42.5)	
≥5	377 (59.4)	355 (57.5)	
PNI status			0.790
No	434 (68.3)	426 (69.0)	
Yes	201 (31.7)	191 (31.0)	
TD count^a^	2 (1-24)	2 (1-38)	0.255

^a^Except these, other values were summarized as frequencies and percentages. CEA: carcinoembryonic antigen; PNI: perineural invasion; TD: tumor deposit.

**Table 2:** Clinicopathologic characteristic of training, validation, and propensity-score-matched cohorts stratified by TD counts.

Characteristic	Training cohort (*n* = 635)			Validation cohort (*n* = 617)			PSM cohort (*n* = 339)		
	1 ≤ count ≤ 3 (*n* = 508)	Counts ≥ 4 (*n* = 127)	*P* value	1 ≤ count ≤ 3 (*n* = 465)	Counts ≥ 4 (*n* = 152)	*P* value	1 ≤ count ≤ 3 (*n* = 188)	Counts ≥ 4 (*n* = 151)	*P* value
Age at diagnosis^a^ (years)	60 (18-75)	57 (21-75)	0.344	60 (26-75)	58 (28-75)	0.274	61 (29-75)	58 (28-75)	0.287
Gender			0.498			0.891			0.889
Male	281 (55.3)	66 (52.0)		254 (54.6)	84 (55.3)		106 (56.4)	84 (55.6)	
Female	227 (44.7)	61 (48.0)		211 (45.4)	68 (44.7)		82 (43.6)	67 (44.4)	
Race			0.802			0.557			0.558
White	399 (78.5)	103 (81.1)		347 (74.6)	120 (78.9)		140 (74.5)	119 (78.8)	
Black	57 (11.2)	12 (9.4)		60 (12.9)	16 (10.5)		21 (11.2)	16 (10.6)	
Other	52 (10.2)	12 (9.4)		58 (12.4)	16 (10.5)		27 (14.4)	16 (10.6)	
Tumor location			0.495			0.506			0.508
Rectum	93 (18.3)	29 (22.8)		86 (18.5)	34 (22.4)		43 (22.9)	34 (22.5)	
Left colon	222 (43.7)	51 (40.2)		221 (47.5)	72 (47.4)		98 (52.1)	71 (47.0)	
Right colon	193 (38.0)	47 (37.0)		158 (34.0)	46 (30.3)		47 (25.0)	46 (30.5)	
Differentiation degree			0.029			0.021			0.239
Well/moderately differentiated	388 (76.4)	85 (66.9)		372 (80.0)	108 (71.1)		145 (77.1)	108 (71.5)	
Poorly differentiated/undifferentiated	120 (23.6)	42 (33.1)		93 (20.0)	44 (28.9)		43 (22.9)	43 (28.5)	
Pathological types			0.461			0.882			0.759
Adenocarcinoma	470 (92.5)	115 (90.6)		427 (91.8)	139 (91.4)		170 (90.4)	138 (91.4)	
Mucinous adenocarcinoma/signet-ring cell carcinoma	38 (7.5)	12 (9.4)		38 (8.2)	13 (8.6)		18 (9.6)	13 (8.6)	
Tumor size (cm)			0.842			0.202			0.762
<5	279 (54.9)	71 (55.9)		251 (54.0)	73 (48.0)		94 (50.0)	73 (48.3)	
≥5	229 (45.1)	56 (44.1)		214 (46.0)	79 (52.0)		94 (50.0)	78 (51.7)	
T stage			0.428			0.163			0.620
T1/T2	29 (5.7)	5 (3.9)		37 (8.0)	7 (4.6)		11 (5.9)	7 (4.6)	
T3/T4	479 (94.3)	122 (96.1)		428 (92.0)	145 (95.4)		177 (94.1)	144 (95.4)	
The number of LNMs			<0.001			<0.001			0.091
0	149 (29.3)	17 (13.4)		151 (32.5)	40 (26.3)		48 (25.5)	40 (26.5)	
1-3	188 (37.0)	48 (37.8)		181 (38.9)	41 (27.0)		71 (37.8)	41 (27.2)	
≥4	171 (33.7)	62 (48.8)		133 (28.6)	71 (46.7)		69 (36.7)	70 (46.4)	
M stage			<0.001			<0.001			0.685
M0	340 (66.9)	60 (47.2)		331 (71.2)	81 (53.3)		105 (55.9)	81 (53.6)	
M1	168 (33.1)	67 (52.8)		134 (28.8)	71 (46.7)		83 (44.1)	70 (46.4)	
CEA level (U/ml)			0.032			0.071			0.962
<5	217 (42.7)	41 (32.3)		207 (44.5)	55 (36.2)		68 (36.2)	55 (36.4)	
≥5	291 (57.3)	86 (67.7)		258 (55.5)	97 (63.8)		120 (63.8)	96 (63.6)	
PNI status			<0.001			<0.001			0.136
No	366 (72.0)	68 (53.5)		340 (73.1)	86 (56.6)		122 (64.9)	86 (57.0)	
Yes	142 (28.0)	59 (46.5)		125 (26.9)	66 (43.4)		66 (35.1)	65 (43.0)	

^a^Except these, other values were summarized as frequencies and percentages. TD: tumor deposit; CEA: carcinoembryonic antigen; PNI: perineural invasion.

**Table 3:** Cox regression analyses predicting DSS in propensity-score-matched cohort.

Characteristic	Univariate analysis		Multivariate analysis	
	HR (95% CI)	*P* value	HR (95% CI)	*P* value
Age at diagnosis (years)	0.999 (0.984-1.014)	0.874		
Gender				
Male	1			
Female	1.182 (0.866-1.615)	0.292		
Race				
White	1			
Black	1.244 (0.782-1.979)	0.357		
Other	0.795 (0.484-1.305)	0.364		
Tumor location				
Rectum	1		1	
Left colon	1.302 (0.842-2.013)	0.236	1.012 (0.647-1.584)	0.957
Right colon	2.378 (1.513-3.739)	<0.001	1.330 (0.839-2.135)	0.237
Differentiation degree				
Well/moderately differentiated	1		1	
Poorly differentiated/undifferentiated	1.640 (1.168-2.302)	0.004	1.682 (1.184-2.388)	0.004
Pathological types				
Adenocarcinoma	1			
Mucinous adenocarcinoma/signet-ring cell carcinoma	1.096 (0.653-1.839)	0.728		
Tumor size (cm)				
<5	1		1	
≥5	1.444 (1.055-1.975)	0.022	1.269 (0.919-1.753)	0.148
T stage				
T1/T2	1		1	
T3/T4	4.296 (1.368-13.493)	0.013	1.468 (0.454-4.792)	0.525
The number of LNMs				
0	1		1	
1-3	1.114 (0.708-1.753)	0.641	0.726 (0.454-1.160)	0.181
≥4	2.041 (1.354-3.076)	0.001	1.099 (0.712-1.696)	0.669
M stage				
M0	1		1	
M1	4.677 (3.319-6.590)	<0.001	3.343 (2.306-4.847)	<0.001
CEA level (U/ml)				
<5	1		1	
≥5	3.787 (2.504-5.727)	<0.001	2.277 (1.444-3.589)	<0.001
PNI status				
No	1		1	
Yes	1.692 (1.239-2.312)	0.001	1.313 (0.951-1.813)	0.098
TD count				
1-3	1		1	
≥4	1.641 (1.201-2.243)	0.002	1.820 (1.320-2.509)	<0.001

HR: hazard ratio; CI: confidence interval; CEA: carcinoembryonic antigen; PNI: perineural invasion; TD: tumor deposit.

## Data Availability

The data used to support the findings of this study are available from the corresponding author upon request.
