# Corrigendum to “The Prognostic Significance of Tumor Deposit Count for Colorectal Cancer Patients after Radical Surgery”

**DOI:** 10.1155/2020/1848252

**Published:** 2020-09-19

**Authors:** Kuo Zheng, Nanxin Zheng, Cheng Xin, Leqi Zhou, Ge Sun, Rongbo Wen, Hang Zhang, Guanyu Yu, Chenguang Bai, Wei Zhang

**Affiliations:** ^1^Department of Colorectal Surgery, Changhai Hospital, Naval Medical University, Shanghai, China; ^2^Department of Pathology, Changhai Hospital, Shanghai, China

In the article titled “The Prognostic Significance of Tumor Deposit Count for Colorectal Cancer Patients after Radical Surgery” [1], the authors “Kuo Zheng, Nanxin Zheng, Cheng Xin, Leqi Zhou, Ge Sun, Rongbo Wen, Hang Zhang, Guanyu Yu, and Wei Zhang” were affiliated to the Department of Colorectal Surgery, Changhai Hospital, Shanghai, China which is incorrect. The correct affiliation for the authors is Department of Colorectal Surgery, Changhai Hospital, Naval Medical University, Shanghai, China. The corrected list of affiliations is shown above.
